# Supplementary material for: The interactome of intact mitochondria by cross-linking mass spectrometry provides evidence for coexisting respiratory supercomplexes
Source: Mol Cell Proteomics. 2017 Dec 8;17(2):216–32. doi: 10.1074/mcp.RA117.000470 (PMC5795388; doi:10.1074/mcp.RA117.000470)
Supplement: Supplemental Data [file supp_RA117.000470_133922_0_supp_23978_fzffwf.zip › spectra_annotation/mito_spectra_annotation/49-1-1-1-2-1.pdf]

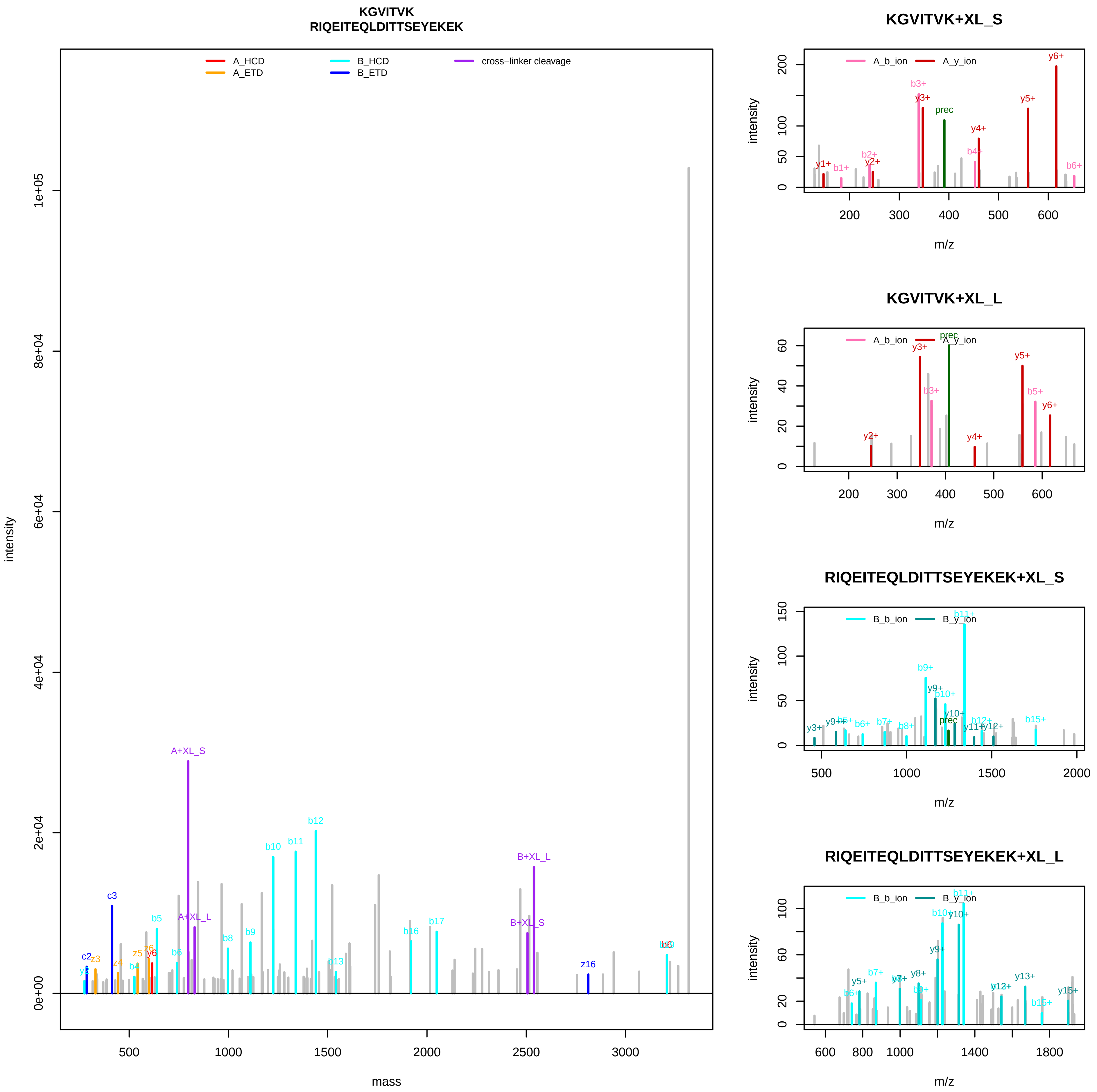
KGVITVK
RIQEITEQLDITTSEYEKEK
A_HCD
A_ETD
B_HCD
B_ETD
cross−linker cleavage
intensity
mass
0e+00
2e+04
4e+04
6e+04
8e+04
1e+05
500
1000
1500
2000
2500
3000
A+XL_S
A+XL_L
B+XL_L
B+XL_S
c2
c3
z3
z4
z5
z6
y6
b4
b5
b6
b8
b9
b10
b11
b12
b13
b16
b17
z16
KGVITVK+XL_S
A_b_ion
A_y_ion
intensity
m/z
prec
y1+
b1+
b2+
y2+
b3+
y3+
y4+
b4+
y5+
y6+
b6+
KGVITVK+XL_L
y5+
b5+
y6+
RIQEITEQLDITTSEYEKEK+XL_S
B_b_ion
B_y_ion
b9+
y9+
b10+
y10+
b11+
y11+
y12+
b12+
b15+
b6+
b7+
b8+
y3+
RIQEITEQLDITTSEYEKEK+XL_L
y5+
b7+
y7+
y8+
y13+
y15+
b15+
y12+
